# External Apical Root Resorption Following Orthodontic Treatment with Clear Aligners Versus Fixed Appliances: A Systematic Review and Meta-Analysis

**DOI:** 10.3390/dj13120580

**Published:** 2025-12-05

**Authors:** Atanaz Darvizeh, José Antonio González Sánchez, Guillermo Doria Jaureguizar, Oriol Quevedo, Fernando de la Iglesia Beyme, Firas Elmsmari, Massimo Del Fabbro

**Affiliations:** 1 Department of Endodontics, School of Dentistry, University of Catalonia (UIC), 08195 Barcelona, Spain; atanazdarvizeh@uic.es (A.D.); jagonzalez@uic.es (J.A.G.S.); guille_doria@uic.es (G.D.J.); 2 Department of Orthodontics, School of Dentistry, International University of Catalonia (UIC), 08195 Barcelona, Spain; uriquevedo@uic.es (O.Q.); fdelaiglesia20@gmail.com (F.d.l.I.B.); 3 Department of Clinical Sciences, College of Dentistry, Ajman University, Ajman P.O. Box 346, United Arab Emirates; 4 Center of Medical and Bio-Allied Health Sciences Research, Ajman University, Ajman P.O. Box 346, United Arab Emirates; 5 Department of Biomedical, Surgical and Dental Sciences, Milan University, 20122 Milan, Italy; 6 Unit of Maxillo-Facial Surgery and Dentistry, Fondazione IRCCS Ca’ Granda Ospedale Maggiore Policlinico, 20122 Milan, Italy

**Keywords:** clear aligners, fixed appliances, meta-analysis, orthodontics treatment, root resorption, systematic review

## Abstract

**Background/Objectives:** Clear aligners (CAs) are a popular alternative to classical fixed appliances (FAs) for orthodontic treatment. This systematic review aimed to compare the external apical root resorption (EARR) in patients undergoing orthodontic therapy with either FAs or removable CAs. **Methods:** An electronic search was conducted to identify comparative studies. Risk of bias was assessed using the Cochrane RoB 2.0 tool for randomized controlled trials (RCTs) and the ROBINS-I tool for non-RCTs. EARR at the following incisors was considered: maxillary central (MxC), maxillary lateral (MxL), mandibular central (MdC), and mandibular lateral (MdL). A random-effects meta-analysis was performed, and mean differences were estimated. **Results:** Ten studies (one RCT, two prospective, and seven retrospective studies) were included. Four had a low risk of bias, four had a moderate risk, and two had a serious concern. In total, 286 patients (1476 incisors) and 289 patients (1487 incisors) in the CA and FA groups were considered, respectively. The mean follow-up was 22.7 ± 9.9 (standard deviation) in the CA group and 22.5 ± 8.2 months in the FA group. The meta-analysis found that CAs caused significantly less EARR than FAs for all tooth types except for MdL. On a patient basis, the mean difference (MD) in favour of CAs ranged from −0.64 mm (95% CI (confidence interval): −0.90, −0.38 mm) for MxC to −0.26 mm (95% CI: −0.43, −0.09 mm) in MdC. Heterogeneity across studies was generally high, except for MdC cases. **Conclusions:** EARR at incisor teeth is generally lower using CAs compared to FAs. Further evidence-based studies are needed to confirm these results and understand the clinical relevance of such a difference.

## 1. Introduction

As orthodontic treatment has advanced over the years, it has offered patients more options to correct malocclusions, particularly with clear aligners (CAs) and fixed appliances (FAs) [1]. However, besides the advantages of orthodontic treatment in malocclusion correction, potential irreversible side effects, such as external apical root resorption (EARR), exist [2]. EARR shortens roots due to orthodontic forces, affecting treatment planning and patient management [3].

**Figure 1:**
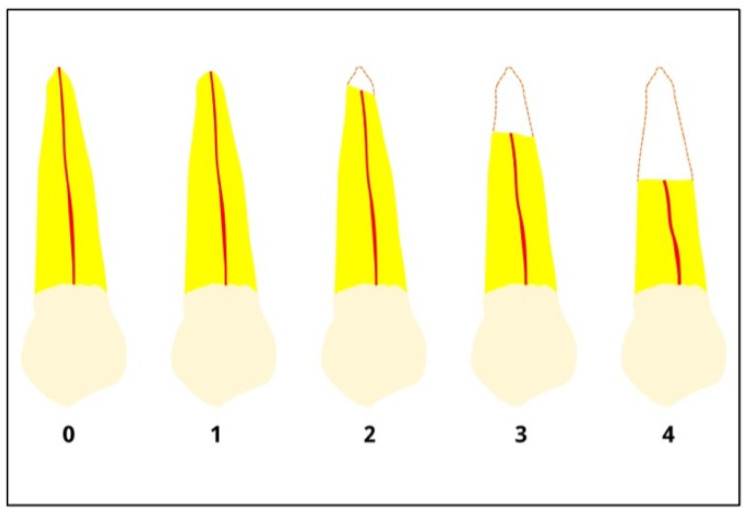
Different degrees of root resorption according to Malmgren et al. [6]. Degree 0: absence of resorption; 1: irregularity in the apical root contour; 2: resorption of up to 2 mm; 3: resorption from 2 mm up to 1/3 of the root; 4: loss greater than 1/3 of the root length. Yellow: root dentin; red line: root canal.

EARR progresses through several stages, each of which may require different management strategies. As shown in Figure 1, the shallow root area ranges from grade 0 for a healthy tooth to grade 4 for significant resorption. Inflammation, infection, periodontal disease, or excessively rapid orthodontic forces may induce this poorly understood process. Gentle, sustained orthodontic forces prevent EARR compared to forceful, short-term forces. Force direction and duration of orthodontic treatment can influence EARR [4,5].

EARR involves the loss of cementum or dentin at the apex, leading to an irreparable reduction in root length, which results in increased tooth mobility and decreased viability [7,8,9,10]. Orthodontic tooth movement concentrates forces on the periodontium, particularly the apical third of the root, leading to a loss of protective cells [11,12] and eventual short- and long-term dental health issues [8,13]. Understanding the occurrence and severity of EARR is essential for assessing treatment outcomes and ensuring patient welfare [14].

CAs have changed orthodontic practice through their aesthetic appeal and patient-friendly features [15], which gently realign teeth using transparent, removable trays, offering a discreet and simplified alternative to braces [16]. New material technology and treatment planning tools allow transparent CAs to fit many malocclusions and patient preferences [17,18].

On the other hand, FAs remain a foundation of orthodontic treatment, owing to their efficacy and versatility, especially in complex multidirectional tooth movement cases [19,20,21].

Studies comparing EARR between CAs and FAs have reported inconsistent results: some find no significant differences [22,23], while others suggest varying EARR patterns [3,14,24,25].

Parameters such as treatment duration, force magnitude, and patient factors may influence the risk of EARR [26].

EARR analysis is challenging due to variations in measuring methods, sample characteristics, and study designs. Both periapical radiography and cone-beam computed tomography (CBCT) measure EARR, but each has limitations and biases; thus, valid comparison studies need standardized measurement [27,28]. Orthodontists must weigh the benefits and drawbacks of each approach based on treatment goals, patient preferences, and EARR susceptibility [29,30].

A meta-analysis evaluates treatment effects and confidence intervals by statistically combining data from multiple studies [31,32]. This strategy improves statistical power and improves estimate precision when studies are too small to properly quantify an intervention’s effect [31,33]. A meta-analysis within a systematic review consolidates effect estimates from multiple studies. It relies on two key models: the fixed-effect model, which assumes a shared effect across studies and focuses only on within-study variation [34], and the random-effects model, recommended when heterogeneity among study effects is high [31].

This systematic review and meta-analysis aimed to compare two orthodontic treatments and their effects on EARR.

## 2. Materials and Methods

The systematic review was conducted in accordance with the Cochrane handbook [35] and the PRISMA 2020 guidelines [36] (see Supplementary PRISMA checklist S1). The review protocol was preliminarily registered in PROSPERO (CRD42024593538). Objectives were set using the PICO framework as follows:

- P (Population): Patients with malocclusion in need of orthodontic treatment, regardless of age or gender.
- I (Intervention): Patients treated with clear aligners.
- C (Control): Patients treated by fixed orthodontic appliances.
- O (Outcome): External apical root resorption in the vertical dimension, measured in mm or % of baseline root length.

Inclusion criteria included human clinical trials reporting EARR in incisor teeth of malocclusion patients undergoing orthodontic treatment with either FAs or CAs, with or without tooth extraction. Randomized studies (RCTs), prospective and retrospective cohort studies with a minimum of 6-month observation period, and at least 10 patients per group were included. No restriction was placed regarding language or publication year.

**Figure 2:**
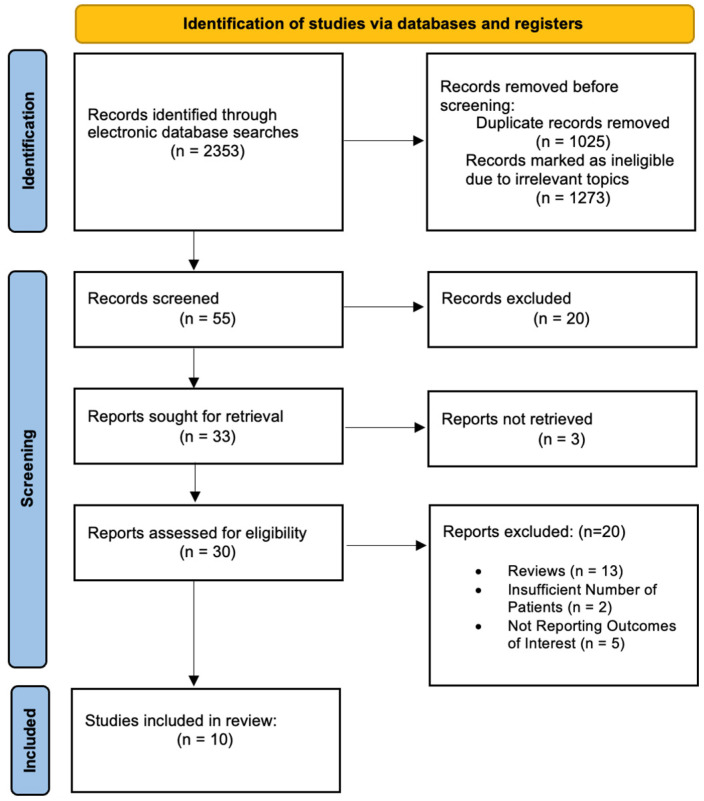
The PRISMA flow diagram.

Exclusion criteria included animal/in vitro studies, meta-analyses, systematic reviews, case reports, non-comparative trials, and abstracts or protocols only (Figure 2, Table S2).

### 2.1. Outcome

The primary outcome was EARR assessed at least six months after beginning the orthodontic treatment, expressed in mm or % as a linear change in root length relative to baseline, and measured through 2D radiographs (intraoral periapical or panoramic radiograph) or 3D techniques (cone-beam computed tomogram).

### 2.2. Search Processing and Data Collection

Database Search: Two reviewers (AD and MDF) conducted a comprehensive search across PubMed/MEDLINE, Embase, Web of Science, LILACS, Scopus, and Cochrane Library, including studies published up to 31 May 2025. Both free-text terms and MeSH terms were used in the search strategy, including clear aligners, external apical root resorption, fixed appliances, malocclusion, orthodontic patients, and orthodontic treatment. Search terms were combined using Boolean operators AND and OR. The search strategy was adapted to each database. An additional search of the grey literature was conducted on Open Grey and Google Scholar. The reference lists of included RCTs and previous systematic reviews were also searched for possible additional eligible studies.

Title and Abstract Screening: Two reviewers (AD and MDF) independently screened articles for relevance, selecting human clinical studies on EARR with CAs and FAs.

Full-Text Retrieval: Full texts of potentially eligible studies were obtained for thorough review before final selection.

Study Selection: Two reviewers independently screened full texts for eligibility to check if studies met the inclusion criteria. Any disagreement was resolved by a third reviewer (JG). Eligible studies meeting the inclusion criteria were included in the qualitative analysis.

Exclusion Reasons: Studies were excluded for irrelevant outcomes, non-comparative designs, insufficient data, mean values and standard deviation (SD) of EARR not provided in mm or %, and duplicate studies.

Data extraction: Data were extracted systematically, including sample sizes, mean EARR values, and SDs for CAs and FAs, as well as study design, treatment duration, and patient demographics. The extracted data were then used for calculating mean differences (MDs) with 95% confidence intervals (CIs) to compare EARR between treatments. In case of missing data, the corresponding authors were contacted by email up to two times. If data were not provided, the study was not included in the quantitative analysis (meta-analysis).

### 2.3. Risk of Bias Assessment

Risk of bias was assessed using the Cochrane Risk of Bias tool (ROB 2.0) [35] for RCTs and the ROBINS-I tool [37] for non-randomized studies. In the ROB 2.0, the following types of bias were considered: bias from the randomization process, bias due to deviations from the intended interventions (assignment to an intervention different from the planned one), bias due to missing outcome data, bias in the outcome measurement, and bias in the selection of the reported results. Each signalling question was scored as “partially yes”, “yes”, “no”, “partially no”, or “not indicated”. The overall risk of bias of a specific trial was judged as “low risk of bias”, “some concerns”, or “high risk of bias”.

In the ROBINS-I tool, seven bias domains are evaluated: bias due to confounding (D1), bias in selection of participants into the study (D2), bias in classification of interventions (D3), bias due to deviations from intended interventions (D4), bias due to missing data (D5), bias in measurement of outcomes (D6), and bias in selection of the reported result (D7). Within each domain, signalling questions are included, to which the response options are generally yes, probably yes, probably no, no, and no information. The response options for the overall risk of bias assessments are the following: low risk (the study can be considered comparable to a well-performed RCT); moderate risk (the study is good but not comparable to a well-performed RCT); serious risk (the study suffers from important issues); critical risk of bias (the study has too many issues and should not be considered in any evidence synthesis); and no information is available to formulate a judgement about risk of bias. Disagreements between reviewers were resolved by discussion or by consulting with a third reviewer.

### 2.4. Quantitative Analysis

Primary outcome data (mean values and standard deviations) from included studies were pooled in pairwise meta-analyses to estimate overall effects. Statistical analyses were performed using Review Manager software (RevMan, version 5.4, Cochrane Collaboration, London, UK, 2020) and STATANow/MP 18.5 for Windows (StataCorp LLC, Lakeway Drive, College Station, TX, USA). A random-effects model was applied to adopt a more conservative approach because significant heterogeneity among studies was expected. Both the overall analysis and analyses per tooth type were run considering the patient as the analysis unit. This was made to reduce variability due to the presence of multiple teeth within each patient, which would also violate the assumption of independence among the samples, introducing imprecision in the effect estimation. Linear EARR measurements in mm were also converted to % root resorption by dividing by mean root length, when possible. However, since this conversion might be affected by non-homogeneity in root length assessment across studies, the analyses regarding EARR percentage were run using standardized mean difference to avoid scale bias. A *p*-value < 0.05 was considered statistically significant.

#### 2.4.1. Heterogeneity Assessment

Heterogeneity testing evaluates the variability in effect sizes across studies. The Q test evaluates the null hypothesis of homogeneity, while the I-squared (I^2^) index measures the percentage of variability due to heterogeneity, ranging between 0% (low) and 100% (high) [38,39]. Heterogeneity can be influenced by publication bias, which is assessed using funnel plots.

#### 2.4.2. Sensitivity Analysis

Sensitivity analysis tests the robustness of findings by examining how results change under different conditions, e.g., excluding studies with a high risk of bias. In case studies where a high risk of bias was identified, sensitivity analysis was run.

#### 2.4.3. Subgroup Analysis

The type of incisor was investigated as a possible source of variation for EARR. The effect of tooth extraction on EARR was also planned to be investigated by subgroup analysis, comparing studies/cases with vs. without extractions. Further subgroup analysis was planned to examine the effect of other factors possibly affecting the outcome, such as the radiographic technique (cone-beam CT vs. panoramic or intraoral radiograph), the follow-up duration, and the patients’ age and gender. While random-effects meta-analysis accounts for heterogeneity, subgroup analysis may help identify its sources by splitting studies based on patient, setting, treatment, and evaluation method characteristics [40].

### 2.5. Assessment of Publication Bias

Publication bias arises when studies with significant findings are more likely to be published, leading to an overestimation of the true effect size. We assessed publication bias using funnel plots and statistical tests such as Egger’s test [41].

### 2.6. Assessment of the Overall Certainty of Evidence

The certainty of the evidence was assessed using the GRADE framework Table S3 (overall EARR in mm and %) and presented using a standard summary of findings table (https://epoc.cochrane.org/resources/epoc-resources-review-authors (accessed on 31 October 2025)).

## 3. Results

The PRISMA flow diagram (Figure 2) illustrates the study selection process of 2353 articles initially retrieved; 30 studies met the inclusion criteria, and 10 studies were finally selected for meta-analysis (Table S4).

In total, 286 patients (1476 incisor teeth) for the CA group and 289 patients (1487 incisors) for the FA group were analyzed. The mean follow-up was 22.7 ± 9.9 months in the CA group (range: 6 to 43.7 months) and 22.5 ± 8.2 months in the FA group (range: 6 to 36.6 months). The studies varied in their reporting of data; some analyzed incisors by quadrant, while others did so by jaw, which requires careful interpretation of sample sizes. Data completeness varied across studies.

### 3.1. Risk of Bias Assessment

**Figure 3:**
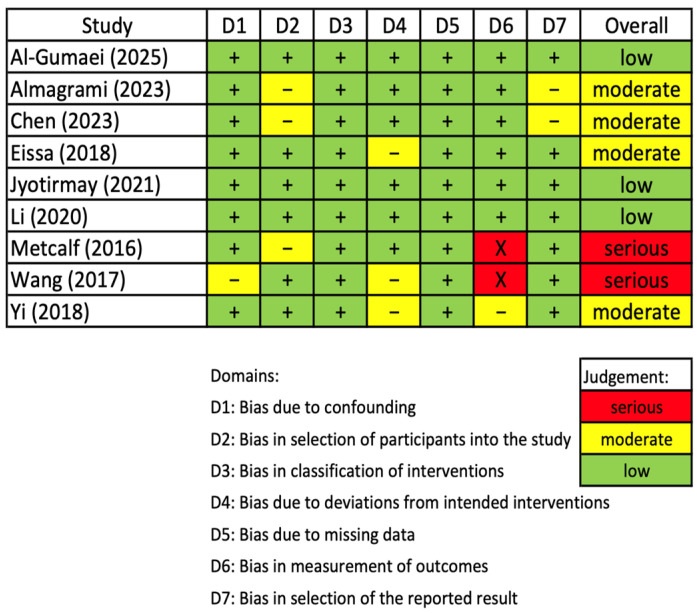
Risk of bias assessment for non-RCT studies using the ROBINS-I tool [3,14,22,24,25,42,43,44,45].

The results of risk of bias assessment for the included non-RCT and RCT studies are presented in Figure 3 and Table 1. Three non-RCT studies [14,24,42] demonstrated an overall low risk of bias, four studies [3,22,25,43] were judged to have a moderate risk, and two [44,45] had a serious concern. The single RCT study [23] was judged as low risk of bias (Table 1).

### 3.2. Meta-Analysis

The results, drawn from 10 studies, provide a concise overview of the available evidence on the topic [3,14,22,23,24,25]. Yi et al. [3] provided results only as the EARR percentage. Therefore, it was not considered for the analysis of EARR in mm.

#### 3.2.1. Overall Results for Incisor Teeth

**Figure 4:**
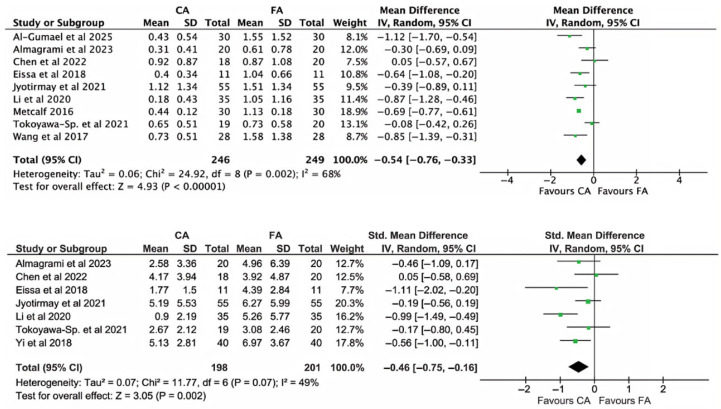
Meta-analysis of EARR considering incisor teeth, evaluated on a patient basis. Top panel: EARR in mm; bottom panel: EARR in %. Both analyses revealed significantly less EARR in the CA group compared with the FA group. Significant heterogeneity was found across studies in the mm-based analysis [3,14,22,23,24,25,42,43,44,45].

Figure 4 illustrates the comparison of EARR (in mm, top panel; in %, bottom panel) between the CA group and FA group for all incisors, considering the patient as the analysis unit. However, it was not possible to calculate the EARR percentage for all studies due to the lack of reported data. Because percentage values had to be estimated in some studies, standardized mean differences were used in the plots on EARR%. For both criteria (mm and %), EARR was significantly lower in the CA group than in the FA group (*p* < 0.00001 and *p* = 0.002 for mm and %, respectively). Heterogeneity was significant only in the mm-based analysis.

#### 3.2.2. Subgroup Analysis per Tooth Type

**Figure 5:**
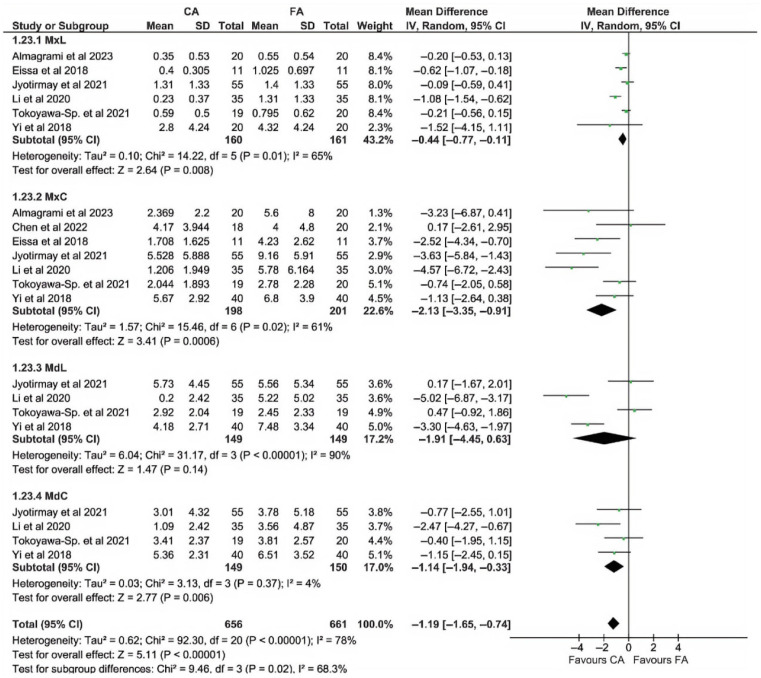
Patient-based subgroup analysis of EARR in mm for different incisor types [3,14,22,23,24,25,43].

**Figure 6:**
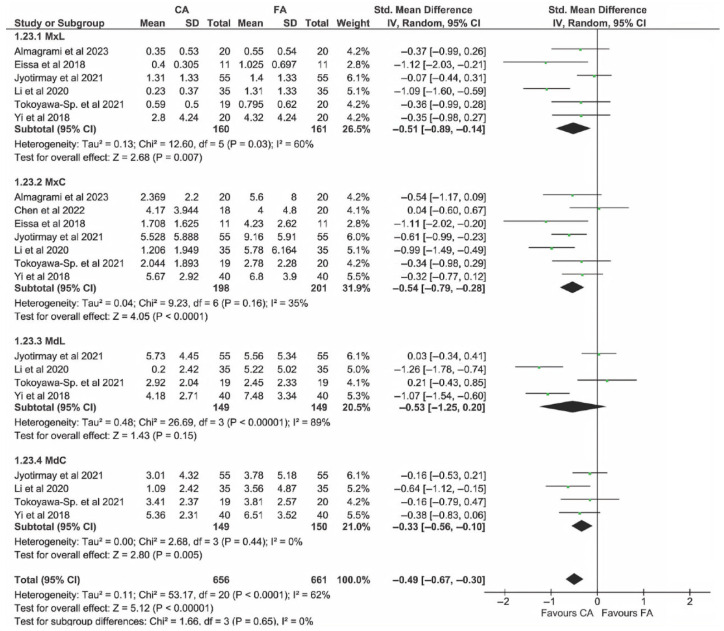
Patient-based subgroup analysis of EARR in % for different incisor types [3,14,22,23,24,25,43].

The results of the per-patient analysis of EARR in mm and %, based on different tooth types considered as subgroups, are depicted in Figure 5 and Figure 6, respectively. The plots indicate significantly lower EARR in the CA group for all incisors, except for MdL. The difference in effects among subgroups was not statistically significant for EARR measured in % (*p* = 0.65), while it was significant when expressed in mm (*p* = 0.02).

As depicted in subgroup analyses of Figure 5 and Figure 6, MdL revealed the highest level of heterogeneity (I^2^ = 90%), for both mm and in %, using patient-level analysis. Lower heterogeneity was generally observed in central incisor subgroups with respect to lateral incisor subgroups.

**Figure 7:**
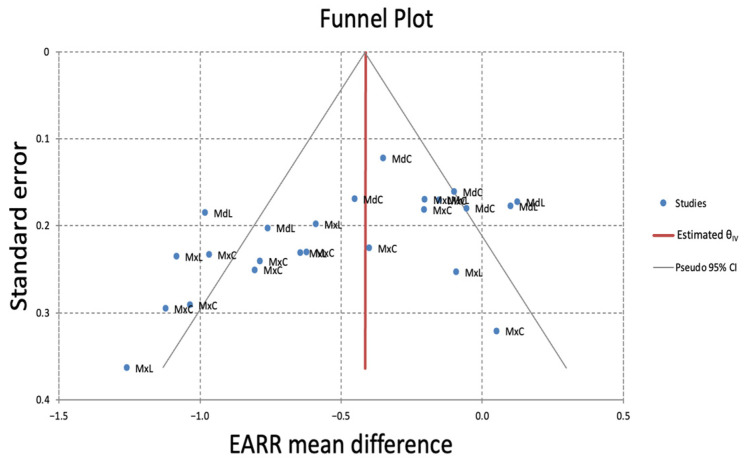
Funnel plot of the meta-analysis of the EARR difference in mm on a patient basis. Subgroup analysis showing the relationship between study-specific standard errors and the EARR mean difference. The vertical red line indicates the overall pooled effect estimate, while the diagonal lines represent the pseudo 95% confidence limits. Visual inspection suggests slight asymmetry, potentially indicating small-study effects or publication bias.

**Figure 8:**
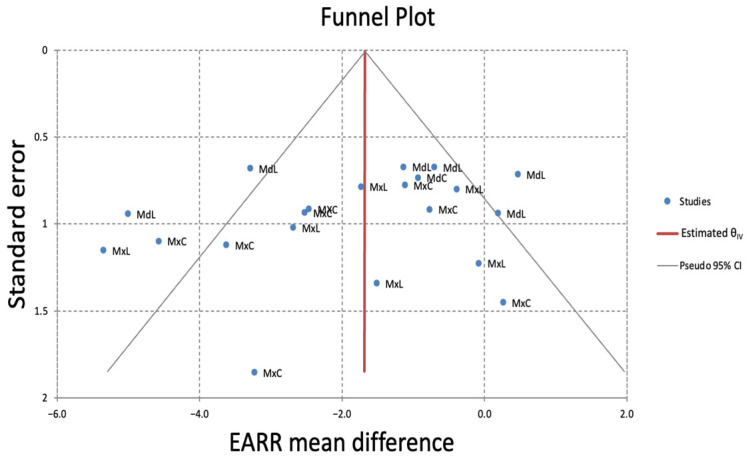
Funnel plot of the meta-analysis of the EARR difference in % on a patient basis. Like Figure 7, visual inspection suggests slight asymmetry, potentially indicating small-study effects or publication bias, mostly attributed to MdL.

The funnel plot for the subgroup analysis of EARR differences in mm, based on patient-level analysis, would suggest moderate publication bias (Figure 7). The MdL teeth were most frequently located outside the 95% CI. Similar results were found for EARR in % (Figure 8). However, since there were fewer than ten studies per outcome, such analyses are underpowered, and results should be interpreted with caution.

#### 3.2.3. Sensitivity Analysis

**Figure 9:**
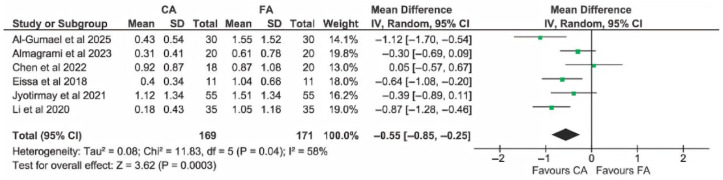
Sensitivity analysis of EARR in mm, evaluated on a patient basis [14,22,24,25,42,43].

Two studies with a high risk of bias were detected [44,45]; therefore, a sensitivity analysis for overall EARR in mm was performed by excluding these studies. Compared with the data shown in Figure 4, the overall effect remained significant (MD: −0.47 mm (95% CI: −0.76, −0.18), *p* = 0.002) in favour of the CA group. Also, heterogeneity across studies remained significant (I^2^ = 66%, *p* = 0.007). A further sensitivity analysis was performed by also excluding the study by Toyokawa-Sperandio et al. [23], because in this study, the follow-up period was shorter than in other studies (only 6 months), and the radiographic technique used was different from that in all other studies, as described in the following subgroup analyses. In this case, exclusion was justified by the different protocol rather than the risk of bias. After the exclusion of such study, as shown in Figure 9, heterogeneity across studies decreased but remained significant (I^2^ = 58%, *p* = 0.04), and the overall effect increased in favour of the CA group (MD: −0.55 mm (95% CI: −0.85, −0.25), *p* = 0.0003).

#### 3.2.4. Subgroup Analysis Based on Tooth Extraction

Only one study (Al-Gumaei et al. [42]) clearly reported outcomes for extraction cases. The other two studies (Li et al. [14] and Jyotirmay et al. [24]) included extraction cases but did not provide separate results for extraction and non-extraction groups. Consequently, this subgroup analysis could not be performed, and no statement can be provided regarding the effect of tooth extraction on EARR due to a lack of evidence.

#### 3.2.5. Subgroup Analysis Based on Follow-Up Time

**Figure 10:**
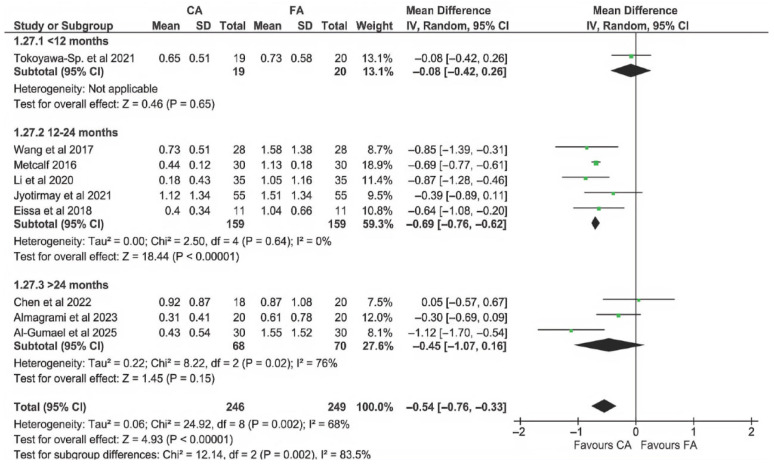
Subgroup analysis comparing studies with different follow-up durations [14,22,23,24,25,42,43,44,45].

Three subgroups were compared (up to 12 months, between 12 and 24 months, and longer than 24 months of follow-up). The analysis (Figure 10) showed that there was a significant effect of follow-up duration among subgroups (*p* = 0.002). This seems to be attributed mainly to the single study with a 6-month follow-up [23], in which no difference in EARR was shown between CAs and FAs. The data in Figure 8 suggest that the advantage associated with CAs starts to become apparent later than 12 months after beginning the treatment. However, due to the few short-term data available, this hypothesis has to be evaluated cautiously. The heterogeneity across subgroups was significant (*p* = 0.002).

#### 3.2.6. Subgroup Analysis Based on Radiographic Technique

**Figure 11:**
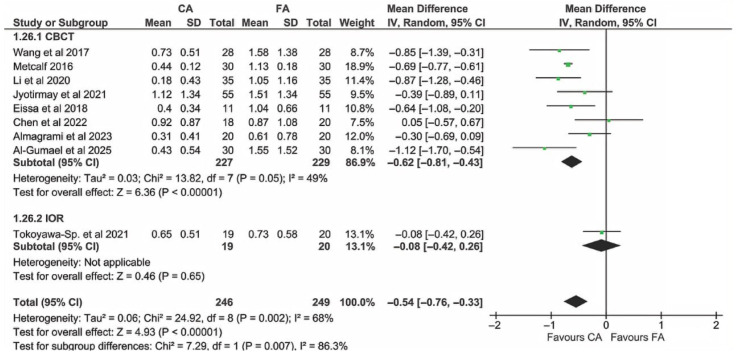
Subgroup analysis comparing studies using different radiographic techniques [14,22,24,25,42,43,44,45].

Two subgroups were compared according to the radiographic technique used for the EARR assessment (CBCT vs. intraoral radiograph (IOR)). Only one study used IOR [23]. The analysis (Figure 11) showed that there was a significant effect of the technique used (*p* = 0.007), with CBCT showing a consistent advantage of CAs over FAs, as opposed to IOR. However, the latter may also be due to the follow-up of the study using IOR, which was only 6 months.

Supplementary Table S4 presents the results of the assessment of the certainty of evidence for overall EARR, as a summary of findings table. For both outcomes, the certainty was scored as low, suggesting that, given the multiple weaknesses of the available evidence, this review only provides some indication of the likely effect. However, the likelihood that the true effect will be substantially different is high.

## 4. Discussion

This work presents a comprehensive review and meta-analysis evaluating EARR in patients treated with CAs vs. FAs. EARR may occur in all teeth, but it most commonly affects maxillary incisors [46]. Only 10 out of 2353 studies initially retrieved met the inclusion criteria, and only 1 was an RCT, indicating a lack of high-quality comparative research, especially on extraction cases. Key questions remain regarding the effectiveness of CAs in reducing EARR and their benefits over FAs. Some studies show that CAs lower EARR risk compared to FAs; for example, Fang et al. [47] reported less EARR for CA groups across all incisors, with a mean difference of 0.65 mm (95% CI: 0.55–0.74 mm, I^2^ = 30%) from three studies. EARR patterns were consistent for the following teeth: MxC (0.79 mm), MxL (0.61 mm), MdC (0.53 mm), and MdL (1.06 mm).

Similarly, Quinatoa et al. [48] found that CAs are associated with lower rates of EARR because of their intermittent forces, which facilitate healing compared to FAs with continuous forces.

Likewise, the systematic reviews by Yassir et al. [49] and Singh et al. [50] found that CAs have a lower risk of EARR compared to FAs.

Moreover, CAs were also connected to decreased resorption in non-extraction cases, according to Elhaddaoui et al. [51]. Also, Zanial et al. [52] found that among eleven studies, six demonstrated a reduced incidence of EARR with CAs, in which a meta-analysis of three studies revealed less resorption by CAs, with an SMD of −0.65 (95% CI: −0.74 to −0.55). However, they did not provide a funnel plot or address the amount of heterogeneity present in the data. Sadauskienė et al. [53] also reported that CAs caused either lowered or similar EARR.

In contrast, Gandhi et al. [54] conducted a systematic review on non-extraction cases and noted no significant difference between the two groups, except for MxL, where CAs showed much reduced resorption. Their methods for analyzing significant root resorption differ substantially from those in this paper. They considered each quadrant of incisors in two subgroups (CAs and FAs), performed a subgroup analysis, and then conducted a comparative analysis for each tooth type.

Our meta-analysis presents the currently available evidence on how CAs and FAs affect EARR in incisor teeth.

Across patient-level analysis, CAs consistently demonstrated lower EARR compared with FAs. Root shortening was significantly less in the CA group, both when measured in millimetres (mean difference of 0.54 mm, *p* < 0.0001) and as a percentage (standardized mean difference of 0.46%, *p* = 0.002). Although moderate heterogeneity was observed across studies, the overall trend highlights a statistically significant advantage of CAs, although the clinical relevance remains uncertain. In fact, such a small difference could be in part ascribed to radiographic measurement variability within and across studies.

Subgroup analyses by tooth type further confirmed this pattern: CAs were associated with significantly less resorption for all incisors except the MdL. For absolute resorption in millimetres, the difference did not reach statistical significance (*p* = 0.08), whereas percentage resorption was significant (*p* = 0.02). The MdL subgroup also displayed the highest heterogeneity (I^2^ = 90%), possibly reflecting anatomical complexity, distinct occlusal forces, or challenges in radiographic assessment. In contrast, central incisors had lower heterogeneity and more reliable results.

Biomechanically, the reduced EARR associated with CAs can be attributed to the nature of the forces they deliver [55]. Clear aligners provide lighter, intermittent forces compared to the continuous forces of FAs, allowing the periodontal ligament more recovery time and minimizing hyalinization and subsequent resorption [56]. The lack of brackets and wires also reduces unwanted tipping or torque, which lowers stress at the root apex. Shorter treatment times often associated with CAs may further enhance this protective effect [57]. Comparable biological responses to orthodontic loading have been observed in alveolar bone, where histological and micro-CT studies show adaptive modelling–remodelling under functional stress. These findings indicate that well-calibrated mechanical forces can promote favourable tissue adaptation while minimizing unwanted resorptive damage [58].

The funnel plot, although based on fewer than ten studies, suggested moderate publication bias, particularly for MdL results, as it repeatedly exceeded the 95% confidence intervals. This may be due to small-study effects, selective reporting, or the failure to publish non-significant findings in certain subgroups.

A notable inadequacy was the lack of analysis of the outcomes based on extraction subgroups. Al-Gumaei et al. [42] was the only study that included extractions, while two others (Li et al. [14] and Jyotirmay et al. [24]) combined extraction and non-extraction cases. Since extractions usually require more space closure and higher force levels, future research should separate data to better assess their impact on EARR.

From a clinical point of view, CAs may be a better treatment choice for patients at higher EARR risk, such as those with short roots or thin alveolar bone or those who were previously orthodontically treated, mainly when central incisors were involved.

It is worth mentioning several limitations of the current study: the included studies did not use the same radiographic evaluation techniques, which may have introduced discrepancies in reported outcomes and variations in measurement precision, thereby contributing to potential errors and increased heterogeneity. Despite a significant *p*-value (*p* = 0.007), the sensitivity analysis in Figure 10 between nine studies using CBCT and only one study using an intraoral radiograph should be considered as exploratory and possibly confounded by the shorter follow-up of that study [23]. Furthermore, due to a lack of standardization across studies, the difference observed might fall within radiographic measurement error. Not all studies reported percentage EARR values or provided root length measurements required for percentage calculation, which limited the consistency of percentage-based analyses. To reduce bias due to the distortion of scales across studies, standardized mean differences were used for the analyses of EARR %. Only one RCT study was available, highlighting the need for further high-quality randomized trials that report outcomes in both millimeters and percentages. The results of funnel plots and Egger’s test for assessment of publication bias may suffer from the low number of studies included and should therefore be interpreted cautiously. Moreover, variations in follow-up duration across studies may have limited the comparability of results. Although age and gender are known to influence susceptibility to root resorption, a subgroup analysis accounting for such variables could not be performed due to the lack of individual data in the included studies. Lastly, because few studies examined MdL teeth or extraction cases, further research might help to better estimate the effect size in these subgroups, considering various factors that may affect EARR. Standardization of radiographic methods, outcome reporting, and follow-up protocols will be essential to strengthen future evidence in this area.

## 5. Conclusions

Clear aligners are linked to a statistically significant reduction in EARR compared to FAs, with the most dependable benefits observed in central incisors. However, also considering the low certainty of evidence resulting from the GRADE assessment, the clinical relevance of this difference remains unclear. Standardized imaging protocols, subgroup reporting by extraction status, and thorough analyses across all incisor types will be crucial to refining these findings and deepening our understanding of the biomechanical and biological mechanisms influencing root resorption.

## Figures and Tables

**Table 1:** Risk of bias assessment for RCTs using the Cochrane 2.0 tool. D1–D5 = domains.

Study	D1	D2	D3	D4	D5	Overall
Toyokawa-Sperandio (2021) [23]	Low	Low	Low	Low	Low	Low

## Data Availability

All data supporting the findings of this study are available in the manuscript.

## Supplementary Materials

The following supporting information can be downloaded at: https://www.mdpi.com/article/10.3390/dj13120580/s1, Table S1. PRISMA checklist. Table S2. Excluded articles as reported in PRISMA Flowchart. Table S3. GRADE summary table: Assessing the certainty of evidence across studies for an outcome (This can also be referred to as ‘quality of the evidence’ or ‘confidence in the estimate’. The “certainty of the evidence” is an assessment of how good an indication the research provides of the likely effect; i.e. the likelihood that the effect will be substantially different from what the research found. By “substantially different” we mean a large enough difference that it might affect a decision). Table S4. Characteristics of included studies in this systematic review and meta-analysis.

## Abbreviations

The following abbreviations are used in this manuscript:

CAs	Clear Aligners
FAs	Fixed Appliances
EARR	External Apical Root Resorption
RCT	Randomized Controlled Trial
MINORS	Methodological Index for Non-Randomized Studies
MxC	Maxillary Central Incisor
MxL	Maxillary Lateral Incisor
MdC	Mandibular Central Incisor
MdL	Mandibular Lateral Incisor
MD	Mean Difference
CI	Confidence Interval
SD	Standard Deviation
PICO	Population, Intervention, Comparison, Outcome
CBCT	Cone-Beam Computed Tomography
PRISMA	Preferred Reporting Items for Systematic Reviews and Meta-Analyses
PROSPERO	International Prospective Register of Systematic Reviews
RoB	Risk of Bias
