# Supplementary material for: External Apical Root Resorption Following Orthodontic Treatment with Clear Aligners Versus Fixed Appliances: A Systematic Review and Meta-Analysis
Source: Dent J (Basel). 2025 Dec 5;13(12):580. doi: 10.3390/dj13120580 (PMC12731763; doi:10.3390/dj13120580)
Supplement: Supplementary file 1 [file dentistry-13-00580-s001.zip › Supplementary Table S2.pdf]

Table S2. Excluded articles as reported in PRISMA Flowchart:

No.	Author/year	Title	Reason for Exclusion
Reviews			
1.	Fang X et al. (2019)	Root resorption in orthodontic treatment with clear aligners: A systematic review and meta-analysis.	Systematic review
2.	Yassir Y.A et al. (2022)	Clinical effectiveness of clear aligner treatment compared to fixed appliance treatment: an overview of systematic reviews.	Systematic review
3.	Singh S et al. (2024)	Comparative assessment of external apical root resorption between subjects treated with clear aligners and fixed orthodontic appliances: a systematic review and meta-analysis.	Systematic review
4.	Elhaddaoui R et al. (2017)	Orthodontic aligners and root resorption: a systematic review	Systematic review
5.	Gandhi V et al. (2021)	Comparison of external apical root resorption with clear aligners and pre-adjusted edgewise appliances.	Systematic review
6.	Al-Zainal M.H et al. (2020)	Clear Aligner Therapy May Not Prevent but May Decrease the Incidence of External Root Resorption Compared to Full Fixed Appliances	Systematic review
7.	Sadauskienė et al. (2020)	Orthodontic treatment with clear aligners and apical root resorption	Systematic review
8.	Alexandra et al. (2023)	Interpretation of the Comparison of Root Resorption Between Dental Aligners and Fixed Appliances by	Systematic review

		Literature Review. Journal of Advanced Zoology	
9.	Inchingolo et al. (2024)	Root Resorption during Orthodontic Treatment with Clear Aligners vs. Fixed Appliances—A Systematic Review	Systematic review
10.	Varoneckaitė et al. (2024)	Comparing Root Resorption in Fixed vs. Clear Aligner Orthodontics: A Radiographic Study	Systematic review
11.	Owolabi N. et al. (2023)	Is Apical Root Resorption Greater When Using Clear Aligners as Compared to Conventional Fixed Appliances in Adult Patients Following Orthodontic Treatment? A literature Review.	Literature Review
12.	Quinatoa et al. (2023)	Interpretation of the Comparison of Root Resorption Between Dental Aligners and Fixed Appliances by Literature Review	Literature Review
13.	Selvaraj M et al. (2025)	Orthodontically induced external apical root resorption with clear aligners compared to fixed appliance treatment: an umbrella review.	Umbrella review
Insufficient Number of Patients			
14.	Khalil et al. (2023)	Apical Root Resorption Accompanied Orthodontic Treatment Using Clear Aligners Versus Fixed Appliances: A Cbct Comparative Study	Included a third group to evaluate Laser effect/low number of patients per group (10 per group)
15.	El-Ghafar et al. (2023)	Clear aligners versus fixed orthodontic appliance on the occurrence of external root resorption in	Insufficient sample for reliable comparison, only cases 14 in total. Moreover, the study assessed only root-volume changes in

		upper teeth; Cone-beam study	mm^3 without linking them to baseline tooth lengths
Not Reporting Outcomes of Interest			
16.	Patil et al. (2022)	Assessment of prevalence of apical root resorption during orthodontic treatment	Presents only pooled prevalence data by sex and tooth type without providing quantitative EARR values
17.	Barbagallo et al. (2008)	Physical properties of root cementum: Comparison of the effects of invisible removable thermoplastic appliances with light and heavy orthodontic forces on premolar cementum. A microcomputed-tomography study	This micro-CT study analysed only premolar before extraction under controlled forces and did not involve full mouth orthodontic treatment
18.	Gonçalves A et al. (2024)	Biological alterations associated with orthodontic treatment with conventional appliances and aligners.	Mixed outcomes, not EARR-specific
19.	Iglesias-Linares A et al. (2017)	Orthodontically induced external apical root resorption in patients treated with fixed appliances vs removable aligners	Genetic factors' effect
20.	Sabban et al. (2024)	Risk of root resorption between Invisalign and fixed orthodontic treatment: A retrospective study	Insufficient quantitative data for meta-analysis
