# Supplementary material for: External Apical Root Resorption Following Orthodontic Treatment with Clear Aligners Versus Fixed Appliances: A Systematic Review and Meta-Analysis
Source: Dent J (Basel). 2025 Dec 5;13(12):580. doi: 10.3390/dj13120580 (PMC12731763; doi:10.3390/dj13120580)
Supplement: Supplementary file 1 [file dentistry-13-00580-s001.zip › Supplementary Table S3.pdf]

Table S3. GRADE summary table: Assessing the certainty[1] of evidence across studies for an outcome

Comparison: clear aligners vs. fixed orthodontics appliances

Certainty assessment of evidence for each outcome

No of studies	Design	Risk of bias	Inconsistency	Indirectness[2]	Imprecision	Other[3]	Relative effect (95% CI)	Certainty (overall score)[4]
	**Outcome:** Overall EARR (external apical root resorption), mm							
9 (total 495 patients)	Randomised trials (1), prospective cohort (2), retrospective (6) (-0.5)	Serious risk of bias (-0.5)	Important inconsistency (-0.5)	No serious indirectness	No serious imprecision	Different follow-up duration; different radiographic techniques (-0.5)	Mean difference -0.54 mm (-0.76, -0.33 mm)	Low (2) ⊕⊕○○
	**Outcome:** Overall EARR (external apical root resorption), %							
7 (total 399 patients)	Randomised trials (1), prospective cohort (1), retrospective (5) (-0.5)	Serious risk of bias (-0.5)	Important inconsistency (-0.5)	No serious indirectness	No serious imprecision	Different follow-up duration; different radiographic techniques (-0.5)	Standardised mean difference -0.46 % (-0.75, -0.16 %)	Low (2) ⊕⊕○○

---

[1] This can also be referred to as 'quality of the evidence' or 'confidence in the estimate'. The "certainty of the evidence" is an assessment of how good an indication the research provides of the likely effect; i.e. the likelihood that the effect will be substantially different from what the research found. By "substantially different" we mean a large enough difference that it might affect a decision.

[2] Indirectness includes consideration of

- Indirect (between study) comparisons
- Indirect (surrogate) outcomes
- Applicability (study populations, interventions or comparisons that are different than those of interest)

[3] Other considerations for downgrading include publication bias. Other considerations for upgrading include a strong association with no plausible confounders, a dose response relationship, and if all plausible confounders or biases would decrease the size of the effect (if there is evidence of an effect), or increase it if there is evidence of no harmful effect (safety)

[4] 4 ⊕⊕⊕⊕ **High** = This research provides a very good indication of the likely effect. The likelihood that the effect will be substantially different** is low.
3 ⊕⊕⊕○ **Moderate** = This research provides a good indication of the likely effect. The likelihood that the effect will be substantially different** is moderate.
2 ⊕⊕○○ **Low** = This research provides some indication of the likely effect. However, the likelihood that it will be substantially different** is high.
1 ⊕○○○ **Very low** = This research does not provide a reliable indication of the likely effect. The likelihood that the effect will be substantially different** is very high.

** Substantially different = a large enough difference that it might affect a decision
