# Supplementary material for: External Apical Root Resorption Following Orthodontic Treatment with Clear Aligners Versus Fixed Appliances: A Systematic Review and Meta-Analysis
Source: Dent J (Basel). 2025 Dec 5;13(12):580. doi: 10.3390/dj13120580 (PMC12731763; doi:10.3390/dj13120580)
Supplement: Supplementary file 1 [file dentistry-13-00580-s001.zip › Supplementary Table S4 .pdf]

Table S4. Characteristics of included studies in this systematic review and meta-analysis

Study	Sample Size	Demographic Information	Orthodontics Treatment Type	Radiographic Registration	Study Design	Outcome<br>Mean of Root resorption ± standard deviation
Yi et al. (2018) [3]	80 patients: total of 640 teeth (320 teeth for each group)	F: 60 M:20<br>average age: 22.54 years<br>CAT: Age 21.80±5.11<br>Female: 31 (77.5%)<br>FOT: Age 23.28±5.60<br>Female: 29 (72.5%)	FOT: preadjusted edgewise appliance with 0.022-in slot<br><br>CAT: sequential thermoplastic appliances	OPG were used to evaluate the root length before and after treatment.<br>All OPGs were taken using the same digital orthopantomography machine (Veraviewepocs, Morita, Kyoto, Japan)	Retrospective	CAT vs. FOT:<br>MxC:<br>5.67±2.92 vs. 6.80±3.90<br>MxL:<br>5.32±3.08 vs. 7.08±3.86<br>MdC:<br>5.36 ±2.31 vs. 6.51±3.52<br>MdL:<br>4.18±2.71 vs. 7.48±3.34
Toyokawa-Sperandio et al. (2021) [23]	40 patients: total of teeth n = 320<br>(20 teeth for each group)	F: 15 M: 25<br>Age (yr):<br>CAT: 23.60±5.65<br>FOT: 20.56±4.51<br><br>CAT:<br>Female: 8(40%)<br><br>FOT:<br>Female: 7(35%)	FOT: fixed metallic orthodontic appliance (slot 0.022 × 0.030, 3M Unitek, Monrovia, CA, USA)<br><br>CAT: SmartTrack, InvisalignTM; (Align Technology, San Jose, CA, USA)	Intraoral radiographs were taken using Dabi Atlante X-ray machine (Dabi Atlante, Ribeirao Preto, Sao Paulo, Brazil) was used (70 kV, 08 mA) with an exposure time of 0.4 seconds	Parallel randomized controlled clinical trial (participants were prospectively recruited and randomized into two groups)	CAT vs. FOT:<br>MxC:<br>11: 0.56±0.44 vs. 0.53±0.50<br>21: 0.52±0.57 vs. 0.86±0.60<br>MxL:<br>12: 0.53±0.51 vs. 0.72±0.52<br>22: 0.65±0.50 vs. 0.87±0.71<br>MdC:<br>31: 0.73±0.62 vs. 0.81±0.60<br>41: 0.85±0.48 vs. 0.88±0.56<br>MdL:<br>32: 0.66±0.45 vs. 0.52±0.59<br>42: 0.77±0.55 vs. 0.66±0.54
Li et al. (2020) [14]	70 patients (total of 373 roots)	F:49 M:21<br>Age (mean ± SD)<br>23.61 ±7.03 years<br>CAT: 24.71 ± 7.48<br>FOT: 22.51 ± 6.47<br>Female:<br>CAT: 22 (63%)<br>FOT: 27 (77%)<br>Extraction (N, %)<br>CAT: 19 (54%)<br>FOT: 14 (40%)	FOT: fixed appliances of Victory (Series; 3 M Unitek, California, USA)<br><br>CAT: Invisalign (Align Technology, California, USA)	CBCT images taken by 3D Accuitomo,<br>Morita Group, JPN with settings of 10 × 10 cm FOV, 85 kV, 4 mA and 360° rotation	Retrospective cohort study	CAT vs. FOT:<br>MxC:<br>0.26±0.42 vs. 1.23±1.31<br>MxL:<br>0.23±0.37 vs. 1.31±1.33<br>Upper canine:<br>0.14±0.53 vs. 1.53±1.92<br>MdC:<br>0.20±0.45 vs. 0.65±0.89<br>MdL:<br>0.04±0.48 vs. 1.02±0.98<br>Lower canine:<br>-0.06±0.47 vs. 1.02±1.33
Jyotirmay (2021) [24]	110 patients (576 roots)<br>A total of 288 roots were analysed in each group	F: 32 M: 23<br>23.71 ± 6.37<br>FOT: 23.71 ± 6.37<br>CAT: 21.62 ± 3.58<br>Females:<br>FOT: 32(59%)<br>CAT: 34 (62%)	FOT: conventional fixed orthodontic appliences<br><br>CAT: not specified clear aligner	CBCT machine (Vatech 3D, Korea) was used for conducting all the CBCT scans	retrospective cohort study	FOT vs. CAT:<br>MxC:<br>2.03 ± 1.31 vs. 1.23 ± 1.31<br>MxL::<br>1.40 ± 1.33 vs. 1.31 ± 1.33<br>Upper Canine:

		Treatment<br>22.45 ± 6.54 vs. 22.23 ± 7.34<br>Cases with extractions<br>26 (47%) vs. 31 (56%)				1.71 ± 1.92 vs. 1.53 ± 1.92<br>MdC:<br>0.65 ± 0.89 vs. 0.55 ± 0.79<br>MdL:<br>1.02 ± 0.98 vs. 1.12 ± 0.87<br>Lower Canine:<br>1.02 ± 1.33 vs. 1.21 ± 1.22
Eissa (2018) [25]	33 patients were included in this pilot study for maxillary incisors.<br>Which is divided in 3 groups;<br>Group I (Smart Track® aligners): 11 patients<br>Group II (Damon brackets): 11 patients<br>Group III (regular brackets): 11 patients.	Group I: F: 6 M:5 mean age of 18.34 ± 2.82 years) with duration 15.14± 1.94 And crowding 5.13±0.59<br>Group III:<br>F:5 M:6<br>mean age of 17.34 ± 2.38 years) with duration 16.27±2.74 and crowding 5.30±0.66	FOT: Damon brackets and regular brackets<br><br>CAT: Smart Track® aligners	CBCT taken before and after orthodontic treatment	Prospective cohort study	Group 1 vs. Group III<br>MxC left:<br>0.46±0.36 vs. 0.97±0.69<br>MxC right:<br>0.51±0.43 vs. 1.14±0.64<br>MxL left:<br>0.44±0.30 vs. 1.09±0.86<br>MxL right:<br>0.36±0.32 vs. 0.96±0.52
Almagrami (2023) [22]	40 patients (160 maxillary incisors)	25.15 ± 6.67 years<br>Age (FOT vs. CAT)<br>22.33 ± 4.33 vs. 25.15 ± 6.67<br>Treatment duration<br>29.67 ± 7.71 vs. 25.85 ± 8.00	FOT: fixed appliances of Victory (Series; 3 M Unitek, California, USA)<br><br>CAT: Invisalign® (Align Technology, California, USA)	CBCT taken by (KaVo® Dental GmbH, Bismarckring, Germany).<br>imaging parameters: voxel size of 120 kV, 5 mA, 0.3 mm and field of view of 230 × 170 mm	retrospective comparative study	FOT vs. CAT:<br>MxC:<br>0.68 ± 0.97 vs. 0.28 ± 0.26<br>MxL:<br>0.55 ± 0.54 vs. 0.35 ± 0.53
Wang et al (2017) [45]	56 patients<br>FOT: M=13, F=15<br>CAT: M=14, F= 14	Age (FOT vs. CAT)<br>15 ± 2.3 vs. 16 ± 3.5<br>Treatment duration<br>1.7 ± 0.4a vs. 1.5 ± 0.3a	FOT: Straight wire appliance (3M Victory Series™)<br><br>CAT: Invisalign® (Align Technology, California, USA)	ERR in millimetre (Cone-beam CT)	Prospective cohort study	FOT:<br>MxC: 1.82 ± 1.45 mm,<br>MxL: 2.24 ± 1.82 mm,<br>MdC: 0.85 ± 0.55 mm<br>MdL: 1.45 ± 0.99 mm<br><br>CAT:<br>MxC: 0.79 ± 0.53 mm,<br>MxL: 0.98 ± 0.62 mm,<br>MdC: 0.50 ± 0.35 mm<br>MdL: 0.69 ± 0.42 mm
Metcalf (2016) [44]	60 patients<br>FOT: M=11, F=19<br>CAT: M=12, F= 18	Age (FOT vs. CAT)<br>19 ± 10.7 vs. 22.2 ± 11.5<br>Treatment duration<br>1.7 ± 0.4a vs. 1.5 ± 0.3a	FOT: pre-adjusted edgewise appliance<br><br>CAT: Invisalign® (Align Technology, California, USA)	ERR in millimetre (Cone-beam CT)	Retrospective radiometric study	FOT:<br>Right MxC: 1.18 ± 1.09 mm,<br>Right MxL: 0.88 ± 0.89 mm,<br>Left MxC: 1.30 ± 1.12 mm<br>Left MxL: 1.17 ± 0.84 mm<br>CAT:<br>Right MxC: 0.33 ± 0.70 mm,<br>Right MxL: 0.36 ± 0.54 mm,<br>Left MxC: 0.58 ± 0.73 mm<br>Left MxL: 0.51 ± 0.74 mm
Chen (2023) [43]	38 patients<br>FOT: 20<br>CAT: 18	Treatment duration (FOT vs CAT)<br><br>CAT: 30.94±4.32 months	FOT: 0.022-in slot Victory Series; 3M Unitek, Calif; Damon Q with a 0.022-in slot-21 (DQ; Ormco, Orange, Calif)	ERR in millimetre (Cone-beam CT)	Retrospective study	MxC:<br>FOT: 0.87 ± 1.08 mm<br>CAT: 0.92 ± 0.87 mm

		FOT: 28.30±5.08 months	CAT: Invisalign® (Align Technology, California, USA)			
Al-Gumaei (2025) [42]	60 patients<br>FOT: 30 (22.97±5.10)<br>CAT: 30 (24.80±5.71)	Treatment duration (FOT vs CAT)<br><br>CAT: 43.70 ± 13.36 months<br>FOT: 36.63 ± 10.40 months	FOT: Metal passive self-ligating fixed braces (0.022 × 0.028 inches; Damon, Ormco, Orange, Calif)<br><br>CAT: Invisalign® (Align Technology, California, USA)	ERR in millimetre (Cone-beam CT)	retrospective comparative study	MxC:<br>FOT: 1.55 ± 1.52 mm<br>CAT: 0.43 ± 0.54 mm

Digital Panoramic Radiographs: OPG
Cone-Beam Computed Tomography: CBCT
CAT: Clear Aligner Treatment
FOT: Fixed Orthodontics Treatment
Maxillary Central Incisor: MxC
Maxillary Lateral Incisors: MxL
Mandibular Central Incisors: MdC
Mandibular Lateral Incisors: MdL
